# Towards a Job Demands-Resources Health Model: Empirical Testing with Generalizable Indicators of Job Demands, Job Resources, and Comprehensive Health Outcomes

**DOI:** 10.1155/2015/959621

**Published:** 2015-10-18

**Authors:** Rebecca Brauchli, Gregor J. Jenny, Désirée Füllemann, Georg F. Bauer

**Affiliations:** Division of Public and Organizational Health, Epidemiology, Biostatistics and Prevention Institute, University of Zurich, Hirschengraben 84, 8001 Zurich, Switzerland

## Abstract

Studies using the Job Demands-Resources (JD-R) model commonly have a heterogeneous focus concerning the variables they investigate—selective job demands and resources as well as burnout and work engagement. The present study applies the rationale of the JD-R model to expand the relevant outcomes of job demands and job resources by linking the JD-R model to the logic of a generic health development framework predicting more broadly positive and negative health. The resulting JD-R health model was operationalized and tested with a generalizable set of job characteristics and positive and negative health outcomes among a heterogeneous sample of 2,159 employees. Applying a theory-driven and a data-driven approach, measures which were generally relevant for all employees were selected. Results from structural equation modeling indicated that the model fitted the data. Multiple group analyses indicated invariance across six organizations, gender, job positions, and three times of measurement. Initial evidence was found for the validity of an expanded JD-R health model. Thereby this study contributes to the current research on job characteristics and health by combining the core idea of the JD-R model with the broader concepts of salutogenic and pathogenic health development processes as well as both positive and negative health outcomes.

## 1. Introduction and Study Aim

In the field of occupational health and safety it is well known that job characteristics affect workers' health and well-being [1, 2]. With the introduction of new working methods and procedures during the 20th century a number of new health and safety hazards at work emerged. Many countries, especially in the European Union, aim to systematically identify factors that lead to occupational health [1]. In the UK, for example, the Health and Safety Executive (HSE) Management Standards Indicator Tool was developed and is increasingly used by organizations to monitor working conditions that can lead to stress [3].

Besides, a lot of research was conducted not only to assess indicators for work-related stress and well-being but also to identify the underlying mechanisms that lead from job characteristics to health and well-being. Among others, very well established is the so-called demand-control model (DCM) [4]; a combination of high job demands and low job control will lead to job strain. An alternative model, the effort-reward imbalance (ERI) model [5], assumes that job strain is the result of an imbalance between effort and reward and may lead to negative health outcomes, such as cardiovascular diseases. However, most studies on the DCM and ERI model have been restricted to a very limited set of independent variables that may not be relevant for all kinds of jobs and persons [6]. To meet this limitation the Job Demands-Resources (JD-R) model [6–8] was developed at the beginning of the century. At its heart lies the assumption that job characteristics can be classified into two general categories: job demands and job resources [6]. Job demands refer to physical, mental, social, or organizational job characteristics that require sustained physical or psychological effort, thus being associated with physiological and/or psychological costs [7]. Job resources refer to those physical, mental, social, or organizational job characteristics that may be functional in meeting job requirements and thus reduce the associated physiological and/or psychological costs and stimulate personal growth and development [6, 7, 9]. The second assumption of the model is that the two categories of job characteristics evoke two relatively independent psychological processes which are considered to play a crucial role in the development of burnout and engagement [6, 10]. The first process—the health impairment process—explains the exhausting impact of job demands, such as poorly designed jobs (e.g., jobs with low job control) or chronic job demands (e.g., work overload or time pressures) on burnout [6, 11]. The second process—the motivational process—suggests that job resources exert a motivating potential and lead to high work engagement. There has been considerable empirical support for these two processes and their impact on burnout and engagement and consequently also on organizational outcomes [12–14]. Further, the JD-R model assumes that job resources are not only related to work engagement but also to burnout, whereas job demands are strongly related to burnout but not or only weakly related to engagement [11, 15].

The JD-R model has been offered as a generic framework to overcome the limited focus of previous stress models such as DCM and ERI model [6]. Thereby it provides broad categories of physical, mental, social, or organizational job characteristics to be included. Since studies using this model are highly diverse regarding the job demands and resources included, it is difficult to compare and aggregate findings regarding key job demands and resources across different studies.

Moreover, in line with the origin of the JD-R model, most studies using this model focus on burnout and engagement as relevant outcomes [6]. An increasing number of studies consider further positive outcomes such as innovativeness, life satisfaction, organizational commitment, perceived health, workability, or happiness and negative outcomes, such as absenteeism, accidents, unsafe behaviors, physical ill health, or turnover intention [15]. However, these studies mostly consider these outcomes as being mediated by work engagement and burnout as suggested by the JD-R model.

To study the direct effect of job characteristics and biopsychosocial health outcomes other than burnout and engagement, a balanced conceptualization of the elements and paths of the JD-R model is needed. Currently, on the predictor side, the model indeed comprehensively acknowledges that both job demands and job resources can be physical, mental, and social in nature [6]. In contrast, the dependent variables do not explicitly consider physical, mental, and social health outcomes but are mostly limited to psychological outcomes, mainly burnout and engagement. Consequently, the paths linking job characteristics to these focused outcomes are also primarily conceptualized as psychological processes (health impairment and motivational process).

## 2. Developing the JD-R Health Model

The present study aims to develop and test a comprehensive JD-R health model with paths linking job demands and job resources to both negative and positive biopsychosocial health outcomes. Thereby we link the JD-R model with the concept of salutogenesis [16] and a generic health development framework applying the conception of positive and negative health [17].

### 2.1. Processes of Health Development: Pathogenesis and Salutogenesis

Antonovsky [16, 18] proposed to complete the concept of pathogenesis, which examines how diseases develop, by the concept of salutogenesis, which explains how health is maintained or strengthened. Antonovsky, who understood health as a “ease-disease-continuum” ranging from minimal to maximal health [16, 18], researched how “Generalized Resistance Resources (GRR)” and “Sense of Coherence (SoC)” contributed to the maintenance of health in potentially harming environments. The health development model [17] links the salutogenic and pathogenic processes by showing how risk factors are related to disease outcomes, whereas resources are related to positive health outcomes. This dual path is in the same line of thinking as general stress research and in particular as the JD-R model: demands that are appraised as threats can lead to a health impairment process—a pathogenic process—straining a person physically, draining him/her mentally, and isolating him/her socially, thus harming his/her self-reproduction. This process can be mitigated by the presence of resources (or “GRR” in Antonovsky's terminology) and SoC, influencing appraisal as well as coping and recovery processes—in the terms of Antonovsky a salutogenic process. Additionally, as the JD-R model has postulated and empirically proven, resources stimulate personal growth and development: A person draws on resources not only to be resilient in the face of potential harmful situations and events, but also to strengthen his/her standing in life and work and to achieve his/her goals. This process leads to a state of energy and vigor, which can be understood in terms of positive health as self-fulfillment. This again puts a person in the position to further build resources and thus protect him/her from negative health outcomes as well as strengthen his positive health status, as research on gain cycles shows [19, 20]. Although Antonovsky's original salutogenic model limits the term salutogenesis to the coping and recovering process as described above, we propose to apply the term also to the process of resources leading to positive health [17].

### 2.2. Negative and Positive Health

Positive and negative health relate to corresponding conceptions spanning more than 60 years, from the preamble of the World Health Organization (WHO) in 1946 [21] to Seligman's proposal for positive health in 2008 [22]. The WHO (1946) defines health as a “(…) state of complete physical, mental and social well-being and not merely the absence of disease or infirmity” (p. 2). Similar definitions have been developed early for mental health [23], subsequently building evidence that human beings can “flourish” emotionally and socially despite mental disorders [24]. Similarly, Seligman [22] proposed a conceptual framework for positive health comprising three categories: subjective (“when a person feels great, defined by high ends of measures of several psychological states”), biological (“the positive ends of physiological function and anatomical structure distributions”), and functional (“how well does the individual function?”). Taken together, health has positive and negative axes, covers physical, mental, and social facets of human life, and has emotional and functional components. Following this, we define negative health as* impaired physical, mental, and social self-reproduction*, which is traditionally linked to medical classification systems. Analogously, we define positive health as* physical, mental, and social self-fulfillment*, for which much research has emerged in recent years (see Journal of Positive Psychology). Examples of negative health are painful musculoskeletal disorders inhibiting movement, anxiety states and depressive mood, and social alienation and exclusion. Examples of positive health are energetic fitness, joy and happiness, and being embedded in harmonious relationships. Clearly, both positive and negative health are interrelated but still have independent characteristics; that is, one can be physically impaired and still mentally and socially fulfilled. Further, these aspects of impaired self-reproduction and of self-fulfillment can be operationalized domain-specific (e.g., joy resulting from work situations) or unspecific (e.g., general happiness in life).

### 2.3. JD-R Health Model

The JD-R health model assumes that job demands directly lead to negative health via a* pathogenic path* whereas job resources directly lead to positive health as well as negative health via a* salutogenic path*. Thereby we assume that job resources have a beneficial impact on negative health since the more resources people have available the easier they recover from demands. In accordance with UK Health and Safety Executive [3] we assume that it is reasonable to identify a generalizable set of indicators (job characteristics) predicting work-related and general health. If we are able to demonstrate the model's stability we increase its public health impact.

From both the perspectives of salutogenesis and occupational health, it would be very interesting to consider individual characteristics such as personal resources (sense of coherence, general and specific self-efficacy, coping skills, optimism, self-esteem, hope, or resilience) potentially relevant for either the salutogenic or the pathogenic process or both in this general model. However, in this study, we focus on* job* demands and* job* resources, thereby excluding personal resources since personal resources can act as moderators, mediators, and/or direct predictors of health [15]. Therefore the integration of personal resources would overload the model conceptually and methodologically.

## 3. Testing the Model: Study Hypotheses

To test this JD-R health model we first identified a common set of indicators of job demands and resources, which are potentially suitable for explaining negative and positive health outcomes within a broad range of occupations and organizations (see Section 4). Second, a set of negative and positive health indicators equally applicable to diverse groups of employees was selected. Based on these generalizable indicators, we (1) tested the model in a heterogeneous sample and (2) validated it for different subgroups—that is, in six different organizations, among female and male employees and among employees with and without managerial function—as well as across time.

### 3.1. Hypothesis 1—Model Testing

We expect to find the dual pathways between (a) job demands and negative health and (b) job resources and positive health. Further, as it is assumed by the JD-R model, we also expect different cross-links between these processes: (c) Job demands and job resources are negatively related. (d) Job resources are negatively related to negative health. (e) Negative and positive health are negatively correlated.

### 3.2. Hypothesis 2—Invariance Testing

We expect that the model holds true for different subgroups, that is, for all of the six organizations, for male as well as for female employees, and for employees with and without managerial function. Further, we expect that it is invariant across time.

## 4. Method

### 4.1. Sample

The present three-wave study with a 1-year time interval used data collected in the context of a large-scale stress management intervention program (see Acknowledgments) implemented between 2008 and 2010 in Switzerland in six medium and large Swiss organizations in diverse business sectors. All members of the organizations were invited to participate. Response rates for the three waves were 70.2%, 64.9%, and 62.3% for t1, t2, and t3, respectively. Analysis of respondents revealed only minor selective dropout in regard to gender (lower for male) and job resources (lower for employees with better resources) [25].

Except for the invariance testing across time, analyses in this study were conducted with the first wave data (t1). This baseline sample consisted of 2,159 employees who worked in six organizations that included three industrial production companies (29.5%; 13.2%; and 18.3%), one food processing company (13.9%), one public administration service (15.3%), and one hospital (9.7%). The sample included 1,392 male (64.5%) and 767 female employees (35.5%), with an average age at t1 of 39.3 years (SD = 11.11). In addition, 42.3% had a higher education degree (college or university). Organizational tenure was 9.0 years (SD = 9.33) with an average of 5.1 years (SD = 6.19) in the present job. The heterogeneity of organizations contributes to significant variations in the study variables such as organizations largely differed concerning, first, their gender ratio (male : female): organization 1: 86.6% : 13.4%; organization 2: 45.3% : 54.7%; organization 3: 91.4% : 8.6%; organization 4: 42.1% : 57.9%; organization 5: 58.5% : 41.5%; organization 6: 17.8% : 82.2%. This ratio clearly depends on the business sector where the organizations operate (see above). Second, they differ concerning the ratio of employees with and without managerial position (yes : no): organization 1: 25.1% : 74.9%; organization 2: 24.3% : 75.7%; organization 3: 47.2% : 52.8%; organization 4: 46.5% : 53.5%; organization 5: 25% : 75%; or organization 6: 28.4% : 71.6%. Finally, the organizations differ concerning the core variables in this study job demands (*M* = 2.53 to *M* = 2.79), job resources (*M* = 3.49 to *M* = 3.98), negative health (*M* = 2.18 to *M* = 2.45), and positive health (*M* = 3.04 to *M* = 3.75).

Participants completed a newly developed online survey (see Acknowledgments) that included questions on work characteristics and health outcomes. Participants were assured of the anonymity of the data in the introduction to the questionnaire. Participation in the survey was on a voluntary basis, with the questionnaire being administered during working time which took about 30 minutes to fill out the basic section of scales (see below). Employees logged into the survey system and received an immediate, automated feedback of their results in form of a “traffic-light” display (red, orange, and green), detailed percentile ranks with regard to benchmark values, and tips for the highlighted topic.

### 4.2. Process of Selection of Scales and Items

In order to identify job demands and resources as well as indicators of negative and positive health which are not organization-specific we followed a stepwise approach: The survey comprised a basic section with 35 validated scales on diverse job demands and resources and a broad range of well-being and health indicators, for which data of all employees were available. On the basis of these 35 scales we first analyzed qualitative information about the presence of various working conditions in the participating companies from the consultants who implemented the stress management intervention in these companies. In this first step these consultants figured as raters (nonparticipative observers) of the qualitative process information which was not accessible via traditional quantitative survey methods as it was implemented in this study. The consultants were asked which negative and positive factors were most salient and relevant within a specific organization during the study. Based on this qualitative information, we excluded specific job demands and job resources restricted to a particular organization (such as physical and environmental demands or client-related issues) and selected those which are expected to be relevant for all the different organizations. Second, we used descriptive statistics (means and standard deviations) to quantitatively identify the most relevant job demands and job resources across all organizations. Finally, using bivariate correlations and principle component analyses, we grouped the work characteristic indicators and excluded scales which did not load distinctively on one factor (such as effort-reward imbalance which is an aggregate rather than a demand in itself). Further, we excluded scales which did not strictly measure a characteristic of the job (such as work-home interference) or loaded negatively on a positive factor (such as social stressors on the social resources factor). This procedure yielded five groups of job demands and job resources: (1) quantitative and (2) qualitative task-related demands, job resources related to (3) manager and (4) peer behavior (support/appreciation), and (5) task-related resources.

We proceeded in a similar way (second step only) with (6) positive and (7) negative health outcomes. Principal component analysis including all scales on health and well-being yielded two factors mainly comprising psychosomatic disorders and psychosocial well-being, respectively. Again, scales loading negatively on a positive factor (such as negative feelings on the positive health factor) were excluded to achieve a maximum of distinctiveness (see also Preparatory Analyses).

### 4.3. Measures

The selection procedure described above resulted in the following measures used for model testing.

*(1) Quantitative Task-Related Job Demands*. Two scales can be subsumed under the factor of quantitative job demands.* Time pressure* and* work interruption* were assessed with four items each ranging from 1 =* very rarely/never* to 5 =* very often/constantly* [26]. An example of an item for time pressure is “At work, how often is a rapid pace of work required?” and for work interruption “How often does it occur that you cannot work on something in peace because something else always comes in between?”

*(2) Qualitative Task-Related Job Demands*. Two scales measured qualitative job demands.* Qualitative overload* is assumed to occur when someone has to fulfill tasks which are too complicated and too difficult. The three items were assessed using a 5-point scale from 1 =* almost never/not at all true* to 5 =* almost always/fully* true [27]. This is a sample item from this scale: “It happens that the work is too difficult for me.”* Uncertainty at work* is characterized by unclear or ambiguous instructions and by the absence of sufficient information to make decisions [26]. This scale uses four items: three on a 5-point scale ranging from 1 =* very rarely/never* to 5 =* very often/constantly* and one item on a 5-point scale from 1 =* from nobody* to 5 =* from more than three persons*. An example item is “From how many people do you regularly receive instructions?” 

*Job Resources Related to (3) Manager and (4) Peer Behavior*. Four scales measure manager behavior; another two scales measure peer behavior.* Supportive leadership* including the degree to which the supervisor is available, the degree to which the supervisor's behavior is respectful and fair, and performance feedback was measured by five items drawn from a questionnaire by Udris and Rimann [27]; for example, “The line manager lets one know how well a job has been done.” This response was scored on a 5-point rating scale from 1 =* almost never/not at all true* to 5 =* almost always/fully true*.* Interpersonal fairness* describes the manner of interpersonal treatment by supervisors during decision-making processes [28]. This scale comprises four items responded on a 5-point scale from 1 =* to a small extent* to 5 =* to a large extent*. An example item is “He/she treated you with respect?”* Manager* and* peer support* were assessed by one item, each drawn from a scale assessing social support received from different persons at work [29]. Participants had to assess how much they can rely on different kinds of people in difficult situations at work, on their direct supervisor and their colleagues among others. Both items were scored on a 5-point scale ranging from 1 =* not true at all* to 5 =* a lot*.* Manager* and* peer appreciation* were also assessed by two single items [30]: “Overall, how satisfied are you with the appreciation of your person shown by your line manager?” and “Overall, how satisfied are you with the appreciation of your person shown by your colleagues?” These items were rated on a 7-point graphical scale using smileys.

*(5) Task-Related Job Resources*. Two scales captured task-related job resources.* Task identity* was assessed by a single item which was “In my job one can produce something or carry out an assignment from A to Z?” rated on a 5-point scale from 1 =* almost never/not at all true* to 5 =* almost always/fully true* [27].* Job control* was assessed using a scale with six items ranging from 1 =* very little/not at all* to 5 =* very much* [26]. An example item is “Can you organize your workday independently?” 

*(6) Negative Health*. Three scales assessed negative health.* Exhaustion* was measured using one dimension of the Oldenburg Burnout Inventory [31]. The eight items of the exhaustion subscale refer to general feelings of emptiness at work, overtaxing from work, a strong need for rest from work, and a state of physical exhaustion through work [7]. An example item is “After my work, I usually feel worn out and weary.” Four answer categories from 1 =* totally disagree* to 4 =* totally agree* were used.* Insomnia* was measured using a short version of the Insomnia Severity Index [32]. The three items covered difficulty in falling asleep, difficulty in staying asleep, and the problem of waking up too early, with one item each. Participants answered the items on a 5-point rating scale from 0 =* none* to 4 =* very*.* Psychosomatic disorders* were measured by seven items concerning headaches, neck or shoulder pain, back pain, pain in the joints and limbs, loss of appetite, stomach disorders, digestion problems, skin problems, and eye problems [33]. Responses to these items were on a 5-point rating scale from 1 =* never* to 5 =* constantly*.

*(7) Positive Health*. Three scales captured positive health.* Work engagement* was measured using the nine-item version of the Utrecht Work Engagement Scale [34], which includes three subscales of three items each:* vigor *(e.g., “At work, I feel I am bursting with energy”),* dedication* (e.g., “My job inspires me”), and* absorption *(e.g., “Time flies when I'm working”). Work engagement was scored on a 7-point scale from 0 =* never* to 6 =* always*.* Job satisfaction *was measured by one item (How satisfied are you with your work in general?) which was rated on a 7-point graphical scale with* smileys* [35].* Affective commitment *was assessed by four items drawn from Allen and Meyer (1990). Commitment was scored on a 7-item rating scale from 1 =* not true at all* to 7 =* almost fully true*. An example item is “I enjoy discussing my organization with people outside it.”* Job-related enthusiasm *was assessed by three items from Warr's [36] measurement of well-being asking whether the participant was optimistic, enthusiastic, and cheerful about the job. They responded on a 5-point scale from 1 =* never* to 5 =* all the time*.

### 4.4. Data Analyses

We tested the hypotheses with structural equation modeling using AMOS 20 software package. The fit of the model was assessed with the *χ* ^2^ statistics, the comparative fit index (CFI), the root mean square error of approximation (RMSEA), and the standardized root mean square residual (SRMR). Values of 0.90 and higher are acceptable for the CFI, whereas values of 0.95 or higher are indicators of an excellent fit [37]. Values of up to 0.08 for the RMSEA and SRMR represent reasonable errors of approximation, whereas values up to 0.05 and 0.01 indicate good and excellent fit, respectively [37, 38].

The invariance of our final model across different organizations, gender, job positions, and time (Hypothesis 2) was tested using multiple group analysis with the AMOS 20 software package. In this procedure, two constrained models (one with equality constraints on regression paths and covariances between latent variables and one additionally with constraints on factor loadings) were compared to a default model without cross group constraints. Traditionally, *χ* ^2^ difference tests are used to assess whether there is a significant difference between the models [39]. However, because *χ* ^2^ is highly dependent on sample size, invariance decisions were based on the differences in CFI, in RMSEA, and in SRMR with a ΔCFI ≤ 0.01 supplemented by a ΔRMSEA ≤ 0.015 and a ΔSRMR ≤ 0.030 indicating invariance [40, 41].

## 5. Results

### 5.1. Preparatory Analyses

Because our study variables were measured via single-source self-reporting, we examined the degree to which common method variance could threat our analyses. Thus, two tests were conducted: first, a Harman single factor test [42] was performed. The examination of the unrotated factor solution indicated the presence of at least six factors; that is, no single factor emerged whereby the first factor explained 30.07%, indicating that common method effects were not a likely contaminant of the results observed in our study. To confirm these results, we additionally controlled for the effects of an unmeasured latent factor in our model [43]. The results indicated that whereas the method factor did improve the model fit, it accounted for a very small proportion (nearly 0%) of the total variance. Both tests suggest that common method variance is not a pervasive problem in this study.

To test the assumed two-factor structure of health (negative and positive health), we split our sample into two random subsamples (*n*'s = 925 and 926) and conducted with one half of the sample an exploratory factor analysis and with the second half a confirmatory factor analysis. For the exploratory part, we conducted a principal component analysis on the seven health scales with oblimin rotation. Two components obtained eigenvalues over Kaiser's criterion of 1, which in combination explained 62.37% of the variance. Besides, the analyses of the scree plot yielded two factors as well. The resulting two factors were as expected (1) sleep disorders, exhaustion, and psychosomatic disorders indicating negative health and (2) job satisfaction, affective commitment, work-related enthusiasm, and work engagement indicating positive health. Next, we conducted a confirmatory factor analysis with the second half of the sample. The two-factor solution we extracted from the exploratory factor analysis was confirmed and showed an acceptable to good model fit (*χ* ^2^(11) = 40.01, *p* < 0.001, CFI = 0.98, and RMSEA = 0.05, SRMR = 0.03) when we allowed the residuals to be correlated between sleep disorders and psychosomatic disorders as well as between affective commitment and enthusiasm. The inclusion of these correlations seems acceptable because the respective variables are drawn from the same scales (see above). Factor loadings ranged between 0.40 (psychosomatic disorders) and 0.89 (exhaustion). Moreover, the covariation between the latent factors representing positive and negative health was −0.48. This solution fitted the data remarkably better than the one-factor solution (*χ* ^2^(12) = 220.57, *p* < 0.001, CFI = 0.86, and RMSEA = 0.14, SRMR = 0.08)

Bivariate correlations and internal consistencies (Cronbach's alpha, where suitable, that is, with exception of the single items) of all study variables are shown in Table 1. Note that all scales were sufficiently reliable.

### 5.2. Model Testing (Hypothesis 1)

First, in order to test Hypothesis 1, the two processes were investigated independently—that is, without cross-links between job demands and job resources as well as between negative and positive health (Model 1). As the results indicated (see Table 2), the model did not fit the data very well since relevant model parameters (*χ* ^2^, df, CFI, and SRMR) were not acceptable. Second, in order to investigate cross-links, job demands and job resources, as postulated by the JD-R model, were allowed to correlate (Model 2). This model showed a superior fit (Δ*χ* ^2^(4) = 695.29; *p* < 0.001).

**Figure 1:**
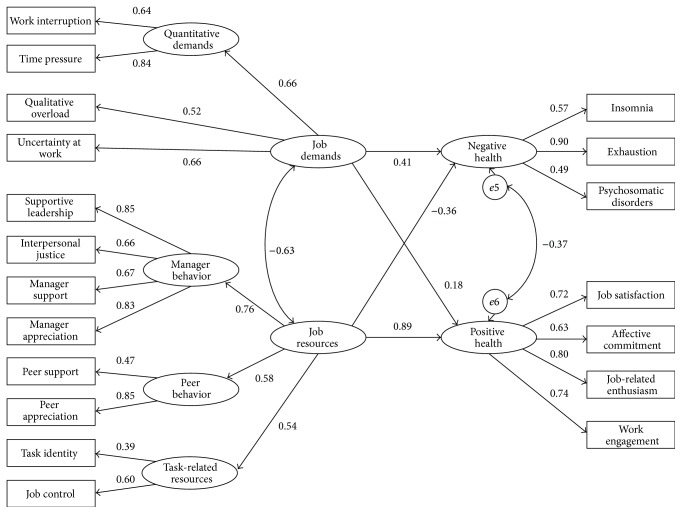
Standardized path coefficients of the final model in the whole sample (*N* = 1,851).

The parameter estimates for the final model are shown in Figure 1. All relations are significant. As expected, the paths from job demands to negative health (*β* = 0.41; *p* < 0.001) and from job resources to positive health (*β* = 0.89; *p* < 0.001) were positive and significant even though the path coefficient from job demands to negative health was lower than the path coefficient from job resources to positive health. Furthermore, the cross-links between job demands and job resources (*β* = −0.63; *p* < 0.001), between job resources and negative health (*β* = −0.36; *p* < 0.001), and, finally, between negative and positive health (*β* = −0.37; *p* < 0.001) were negative and significant as expected. Thus Hypothesis 1 was supported. Moreover, in this model, a total variance of 42.4% (*r* ^2^ = 0.424) in negative health and of 60.2% (*r* ^2^ = 0.602) in positive health is explained by job demands and job resources.

### 5.3. Invariance Testing (Hypothesis 2)

To cross validate the findings, several multiple group analyses were conducted in order to test the assumed invariance of the final model (Hypothesis 2). For each of these analyses, the regression paths and covariances between the latent variables in our model were constrained to be equal across groups. This constrained model (Model 2) was compared with the free model (default model), in which parameter estimates were allowed to vary freely across groups. Next, in addition to constraining the regression paths between the latent variables, the factor loadings were constrained to be equal across groups (Model 3) and this model was compared with the free model.

Across organizations, results of invariance testing showed that both regression paths between the latent variables and the factor loadings were invariant as CFI, RMSEA, and SRMR difference tests (ΔCFI = 0.002 and 0.009; ΔRMSEA = 0.001 and 0.002; ΔSRMR = 0.001 and 0.006) showed (see Table 3).

Also across gender as well as across job positions (managers versus employees) regression paths and covariances between latent variables and factor loadings turned out to be invariant (gender: ΔCFI = 0.001 and 0.003; ΔRMSEA = 0.004 and 0.003; ΔSRMR = 0.001 and 0.000; job position: ΔCFI = 0.001 and 0.001; see Table 4; ΔRMSEA = 0.002 and 0.003; ΔSRMR = 0.001 and 0.004; see Tables 4 and 5).

Finally, results of multiple group testing as displayed in Table 6 indicate invariance across the three measurement points (ΔCFI = 0.000 and 0.000; ΔRMSEA = 0.001 and 0.002; ΔSRMR = 0.004 and 0.002). Thus, results support Hypothesis 2.

Taken together these findings lend support to the proposed expanded JD-R health model explaining negative and positive health. The hypothesized model was confirmed (Hypothesis 1) and could be cross validated across six different organizations, across gender, across job positions, and, finally, across time (Hypothesis 2).

## 6. Discussion

The aim of the present study was to develop and test an expanded JD-R health model with a comprehensive set of job characteristics generalizable to diverse organizations. Thereby we integrate the concept of salutogenesis [16] and a generic health development framework applying the conception of positive and negative health [17].

The resulting model links job demands and resources through two broad paths—a pathogenic as well as a salutogenic path—to both positive and negative biopsychosocial health outcomes. The study could build on a broad range of job demands and resources as well as health outcomes collected within a large-scale stress management intervention study. This allowed to apply qualitative and quantitative methods to empirically select and group global job demands and resources which were relevant for all employees: quantitative and qualitative task-related job demands, job resources related to supportive and appreciative manager and peer behavior, respectively, and task-related resources.

By testing our hypotheses with structural equation modeling, we found evidence for the validity of an expanded JD-R health model predicated on a broad and heterogeneous sample. Invariance testing indeed showed that the model seems to be generalizable to diverse organizations and occupations and, moreover, time invariant. Namely, multiple group analyses indicated invariance across six organizations of various business sectors, across gender and job positions, and, finally, across three times of measurement.

We were able to support the two different pathogenic and salutogenic processes in analogy but expansion of the health impairment and motivational processes of the JD-R model [6, 10]. Our expanded model also supported the cross-links also predicted by the JD-R model [15]. First, we found a strong negative relationship between job demands and job resources, which is in line with other studies [12, 44]. Second, there is a strong and significant negative cross-link between job resources and negative health, and, third, there is a negative, even though not particularly strong, relationship between negative and positive health. These results also correspond with findings from other studies using the JD-R model as a theoretical framework [8, 45].

Furthermore, results showed that the regression weight of the path from job demands to negative health is weaker than that of the path from job resources to positive health. This difference might be explained by the fact that the positive health indicators are closer to the working situation whereas negative health indicators such as insomnia or psychosomatic disorders are more general and influenced by many factors apart from work characteristics (see below). As regards our final goal, namely, to develop a model that included measures of both positive and negative health, the available health indicators were limited in various ways. With the exemption of work-related exhaustion, negative health was assessed in a general way by looking at insomnia and psychosomatic disorders, which are only partially influenced by the work situation. On the other hand, positive health was mainly assessed by work-related measures, which plausibly showed stronger relationships with job characteristics (instead of global indicators such as life satisfaction). In the future, negative and positive health should be assessed equally work-specific or generic to produce more comparable results for the pathogenic and the salutogenic process of the JD-R health model. Specifically, either a narrower study should be undertaken on the side of negative health (i.e., more work-related) or a broader one on the side of positive health (i.e., less work-related). The latter approach could have the advantage of showing the general significance and public health relevance of job characteristics and their beneficial and adverse combination, respectively.

Moreover, the measurement of work characteristics and health was based on self-reported data, which increases the possibility that the relationships between job demands and job resources as well as between negative and positive health could be due to common method variance. Despite the lack of method variance found, there still remains the possibility that this effect may differ across models. Therefore, future research efforts need to consider using multiple methods and measures to eliminate the effect of this potential bias.

Even though we tested time invariance using three times of measurement, this study is cross-sectional. Longitudinal studies with specific research questions and explicit hypotheses concerning change over time would complement the insights of this study [25].

Another limitation concerns the process how we identified the indicators of job demands, job resources, and negative and positive health. This process was explorative and its systematic should be improved, for example, via professional judges or raters instead of consultants.

A third limitation is the lack of a positive impression scale included in the questionnaire in order to exclude that participants answered in a way that will be viewed positively by others which might have biased the results [46].

Fourth, emerging issues (such as stress caused by the economic crisis Europe is still facing) were not captured. It would be very interesting to include such highly relevant concepts in a follow-up study.

Moreover, in this study, we only focused on* job* demands and* job* resources as predictors of health even though it would be interesting to investigate the role of individual characteristics such as personal resources in our model [47]. More studies are needed to reveal the influence of personal resources such as self-efficacy or resilience on the interplay between job characteristics and health.

Finally, in order not only to control for the different organizations but also to specifically investigate the influence the organizational context might have on the variables included in our model as well as the relationships between them, further studies applying a multilevel approach will be needed.

## 7. Conclusions and Contributions

The present study made a step towards an expanded JD-R health model tested with a common set of indicators of job demands and job resources predicting a broad concept of health, including physical, mental, and social health. It contributes to the current research on job characteristics and employees' health by expanding the JD-R model towards a pathogenic and salutogenic path with both negative and positive health outcomes. Building on the JD-R model and on a broad data set with three times of measurement, the present study combined both a theory-driven, deductive approach and a data-driven, inductive approach for the selection of common indicators. We regard our generalized JD-R health model as a contribution to the integration of indicator-focused, evidence-based risk (and resource) assessments within comprehensive frameworks, as was called for by Clausen et al. [48]. We aimed to make the JD-R model comprehensible and useful to researchers with biomedical training by showing that its logic can be expanded from the original health impairment as well as motivational processes to simultaneously study pathogenic and salutogenic health development processes at work. At the same time, the study aimed to show for the first time that this model—operationalized with a manageable number of validated indicators—is stable over time and applicable to diverse economic sectors and professional groups. Therefore, this model is highly useful to show to a broader community who is concerned with public health issues how health-related good psychosocial working conditions are. Moreover, the findings of this study not only showed the relevance of this topic but also can indicate which issues should be addressed when implementing successful population-based public health interventions in the working population.

## Figures and Tables

**Table 1:** Internal consistencies (Cronbach's alphas in italics on the diagonal) and correlations among the variables.

		1	2	3	4	5	6	7	8	9	10	11	12	13	14	15	16	17	18	19
1	Work interruption	*0.805 *																		
2	Time pressure	0.54^∗∗^	*0.829 *																	
3	Qualitative overload	0.18^∗∗^	0.25^∗∗^	*0.788 *																
4	Uncertainty at work	0.34^∗∗^	0.37^∗∗^	0.35^∗∗^	*0.733 *															
5	Supportive leadership	−0.13^∗∗^	−0.17^∗∗^	−0.15^∗∗^	−0.34^∗∗^	*0.816 *														
6	Interpersonal justice	−0.05^∗^	−0.10^∗∗^	−0.16^∗∗^	−0.30^∗∗^	0.57^∗∗^	*0.813 *													
7	Manager support	−0.09^∗∗^	−0.11^∗∗^	−0.12^∗∗^	−0.29^∗∗^	0.59^∗∗^	0.45^∗∗^	—												
8	Manager appreciation	−0.10^∗∗^	−0.15^∗∗^	−0.16^∗∗^	−0.33^∗∗^	0.69^∗∗^	0.55^∗∗^	0.56^∗∗^	—											
9	Peer support	−0.07^∗∗^	−0.05^∗^	−0.09^∗∗^	−0.09^∗∗^	0.16^∗∗^	0.18^∗∗^	0.35^∗∗^	0.12^∗∗^	—										
10	Peer appreciation	−0.09^∗∗^	−0.11^∗∗^	−0.15^∗∗^	−0.16^∗∗^	0.28^∗∗^	0.22^∗∗^	0.15^∗∗^	0.39^∗∗^	0.37^∗∗^	—									
11	Task identity	−0.09^∗∗^	−0.06^∗^	−0.10^∗∗^	−0.15^∗∗^	0.12^∗∗^	0.09^∗∗^	0.09^∗∗^	0.13^∗∗^	0.12^∗∗^	0.14^∗∗^	—								
12	Job control	0.11^∗∗^	−0.03	−0.14^∗∗^	−0.15^∗∗^	0.20^∗∗^	0.25^∗∗^	0.13^∗∗^	0.22^∗∗^	0.04	0.14^∗∗^	0.23^∗∗^	*0.859 *							
13	Insomnia	0.13^∗∗^	0.19^∗∗^	0.24^∗∗^	0.20^∗∗^	−0.20^∗∗^	−0.22^∗∗^	−0.16^∗∗^	−0.21^∗∗^	−0.17^∗∗^	−0.19^∗∗^	−0.11^∗∗^	−0.18^∗∗^	*0.709 *						
14	Exhaustion	0.24^∗∗^	0.40^∗∗^	0.35^∗∗^	0.31^∗∗^	−0.32^∗∗^	−0.26^∗∗^	−0.22^∗∗^	−0.31^∗∗^	−0.15^∗∗^	−0.24^∗∗^	−0.12^∗∗^	−0.24^∗∗^	0.51^∗∗^	*0.820 *					
15	Psychosomatic disorders	0.15^∗∗^	0.19^∗∗^	0.21^∗∗^	0.18^∗∗^	−0.17^∗∗^	−0.21^∗∗^	−0.14^∗∗^	−0.18^∗∗^	−0.13^∗∗^	−0.18^∗∗^	−0.08^∗∗^	−0.17^∗∗^	0.43^∗∗^	0.44^∗∗^	*0.740 *				
16	Job satisfaction	−0.16^∗∗^	−0.18^∗∗^	−0.20^∗∗^	−0.30^∗∗^	0.47^∗∗^	0.38^∗∗^	0.33^∗∗^	0.53^∗∗^	0.14^∗∗^	0.36^∗∗^	0.14^∗∗^	0.24^∗∗^	−0.27^∗∗^	−0.43^∗∗^	−0.24^∗∗^	—			
17	Affective commitment	−0.01	0.02	−0.16^∗∗^	−0.19^∗∗^	0.27^∗∗^	0.20^∗∗^	0.17^∗∗^	0.26^∗∗^	0.04	0.18^∗∗^	0.11^∗∗^	0.19^∗∗^	−0.11^∗∗^	−0.24^∗∗^	−0.12^∗∗^	0.45^∗∗^	*0.817 *		
18	Job-related enthusiasm	−0.10^∗∗^	−0.13^∗∗^	−0.19^∗∗^	−0.22^∗∗^	0.36^∗∗^	0.25^∗∗^	0.24^∗∗^	0.39^∗∗^	0.14^∗∗^	0.30^∗∗^	0.09^∗∗^	0.18^∗∗^	−0.30^∗∗^	−0.48^∗∗^	−0.25^∗∗^	0.56^∗∗^	0.39^∗∗^	*0.836 *	
19	Work engagement	−0.04	0.03	−0.17^∗∗^	−0.17^∗∗^	0.27^∗∗^	0.18^∗∗^	0.15^∗∗^	0.29^∗∗^	0.09^∗∗^	0.25^∗∗^	0.12^∗∗^	0.22^∗∗^	−0.27^∗∗^	−0.39^∗∗^	0.22^∗∗^	0.487^∗∗^	0.53^∗∗^	0.61^∗∗^	*0.942 *

*Note*. *N* = 1,851. Cronbach's alphas appear on the diagonals where appropriate.  ^∗^ *p* ≤ 0.05,  ^∗∗^ *p* ≤ 0.01 (two-tailed).

**Table 2:** Fit statistics for alternative models (model-testing).

Model	df	*χ* ^2^ (*N* = 1,850)	*χ* ^2^/df	GFI	CFI	RMSEA	SRMR	Δ*χ* ^2^
1 (without cross-links)	142	1784.96	12.57	0.91	0.86	0.08	0.13	—
2 (final model)	138	1089.67	7.90	0.94	0.92	0.06	0.05	695.29^∗∗∗^

*Note*. GFI = goodness-of-fit index; CFI = comparative fit index; and RMSEA = root mean square error of approximation.

^∗∗∗^ *p* < 0.001.

**Table 3:** Fit statistics for multigroup analyses and invariance tests across organizations (*N*_Org.1_ = 543, *N*_Org.2_ = 247, *N*_Org.3_ = 247, *N*_Org.4_ = 284, *N*_Org.5_ = 337, and *N*_Org.6_ = 169).

Model	df	*χ* ^2^	*χ* ^2^/df	GFI	CFI	RMSEA	SRMR	Δ*χ* ^2^	Δdf	ΔCFI	ΔRMSEA	ΔSRMR
Organizations												
Model 1 (default model)	342	1025.48	3.00	0.92	0.90	0.03	0.06	—	—	—	—	—
Model 2 (regression paths and covariances between latent variables constrained to be equal across groups)	372	1072.89	2.88	0.92	0.90	0.03	0.06	47.41^∗^	30	0.002	0.001	0.001
Model 3 (Model 2 and factor loadings constrained to be equal across groups)	417	1163.2	2.79	0.91	0.89	0.03	0.07	137.72^∗∗∗^	75	0.009	0.002	0.006

*Note*. GFI = goodness-of-fit index; CFI = comparative fit index; RMSEA = root mean square error of approximation; and SRMR = standardized root mean square residual.

^∗^ *p* < 0.05,  ^∗∗^ *p* < 0.01, and  ^∗∗∗^ *p* < 0.001.

**Table 4:** Fit statistics for multigroup analyses and invariance tests across gender (*N*_male_ = 1,200, *N*_female_ = 651).

Model	df	*χ* ^2^	*χ* ^2^/df	GFI	CFI	RMSEA	SRMR	Δ*χ* ^2^	Δdf	ΔCFI	ΔRMSEA	ΔSRMR
Organizations												
Model 1 (default model)	114	790.06	6.93	0.94	0.90	0.06	0.06	—	—	—	—	—
Model 2 (regression paths and covariances between latent variables constrained to be equal across groups)	120	799.47	6.66	0.94	0.90	0.05	0.06	9.41 (*p* = 0.152)	6	0.001	0.004	0.001
Model 3 (Model 2 and factor loadings constrained to be equal across groups)	129	822.26	6.37	0.94	0.90	0.05	0.05	32.20^∗∗^	15	0.003	0.003	0.000

*Note*. GFI = goodness-of-fit index; CFI = comparative fit index; RMSEA = root mean square error of approximation; and SRMR = standardized root mean square residual.

^∗^ *p* < 0.05,  ^∗∗^ *p* < 0.01, and  ^∗∗∗^ *p* < 0.001.

**Table 5:** Fit statistics for multigroup analyses and invariance tests across job position (*N*_managers_ = 600, *N*_employees_ = 1,250).

Model	df	*χ* ^2^	*χ* ^2^/df	GFI	CFI	RMSEA	SRMR	Δ*χ* ^2^	Δdf	ΔCFI	ΔRMSEA	ΔSRMR
Organizations												
Model 1 (default model)	114	770.41	6.758	0.94	0.90	0.06	0.06	—	—	—	—	—
Model 2 (regression paths and covariances between latent variables constrained to be equal across groups)	120	774.79	6.457	0.94	0.90	0.05	0.06	4.38 (*p* = 0.625)	6	0.001	0.002	0.000
Model 3 (Model 2 and factor loadings constrained to be equal across groups)	129	792.065	6.14	0.94	0.90	0.05	0.06	21.655 (*p* = 0.117)	15	0.001	0.003	0.004

*Note*. GFI = goodness-of-fit index; CFI = comparative fit index; RMSEA = root mean square error of approximation; and SRMR = standardized root mean square residual.

^∗^ *p* < 0.05,  ^∗∗^ *p* < 0.01, and  ^∗∗∗^ *p* < 0.001.

**Table 6:** Fit statistics for multigroup analyses and invariance tests across time (*N*_*t*1_ = 1,858, *N*_*t*2_ = 1,913, and *N*_*t*3_ = 1,754).

Model	df	*χ* ^2^	*χ* ^2^/df	GFI	CFI	RMSEA	SRMR	Δ*χ* ^2^	Δdf	ΔCFI	ΔRMSEA	ΔSRMR
Organizations												
Model 1 (default model)	414	3147.67	7.60	0.94	0.93	0.04	0.05	—	—	—	—	—
Model 2 (regression paths and covariances between latent variables constrained to be equal across groups)	432	3178.18	7.36	0.94	0.93	0.03	0.06	30.51^∗^	16	0.000	0.001	0.004
Model 3 (Model 2 and factor loadings constrained to be equal across groups)	456	3220.44	7.06	0.94	0.92	0.03	0.05	72.77^∗∗∗^	42	0.000	0.002	0.002

*Note*. GFI = goodness-of-fit index; CFI = comparative fit index; RMSEA = root mean square error of approximation; and SRMR = standardized root mean square residual.

^∗^ *p* < 0.05,  ^∗∗^ *p* < 0.01, and  ^∗∗∗^ *p* < 0.001.
